# Coexistence of Chronic Lymphocytic Leukemia and Myeloproliferative Neoplasm

**DOI:** 10.1155/2014/512928

**Published:** 2014-09-02

**Authors:** Patrycja Zielinska, Miroslaw Markiewicz, Monika Dzierzak-Mietla, Anna Koclega, Grzegorz Helbig, Slawomira Kyrcz-Krzemien

**Affiliations:** Department of Hematology and Bone Marrow Transplantation, Medical University of Silesia, Dabrowskiego 25, 40-032 Katowice, Poland

## Abstract

Chronic lymphocytic leukemia (CLL) is the most common adult leukemia in the Western world. Host immune surveillance caused mainly by the disease itself is speculated to be responsible for high incidence of secondary neoplasms. However, the simultaneous occurrence of CLL and myeloproliferative disorder in the same patient is extremely rare. In the present report, a case of an 81-year-old man who was diagnosed with chronic lymphocytic leukemia and concomitant essential thrombocythemia is presented. We describe the morphologic, immunophenotypic, cytogenetic, and molecular findings in this patient. We also review the current literature.

## 1. Introduction

Chronic lymphocytic leukemia (CLL) is the most common adult leukemia one may encounter in the Western world. High incidence of secondary neoplasms (solid tumors and hematological neoplasms) has been reported before [1, 2]. There has been described a broad spectrum of secondary solid tumors in CLL patients, for example, carcinomas of stomach, colon, breast, and kidney [1, 2]. Also different hematological malignancies, like myeloproliferative neoplasms (MPNs), were reported to occur in CLL patients before or during the course of the disease [1]. The simultaneous occurrence of lymphoproliferative and myeloproliferative disorder is very rare [1–4]. It is still under investigation whether these two clonal disorders are caused by one primary genetic alteration that increased genomic instability and susceptibility to further clonal disorders or are purely coincidental findings of a different origin [5–8].

## 2. Case Report

We report a case of an 81-year-old Caucasian man who was referred to our Department of Hematology and Bone Marrow Transplantation, Medical University of Silesia, Katowice, Poland, due to elevated platelet count up to 2900 × 10^9^/L. The patient has consented to this publication.

The patient presented with a 6-month history of fatigue, gradual weight loss, and dyspnea on exertion. Before admission to our department the patient was hospitalized in the municipal hospital. His blood test revealed elevated platelet (PLT) count up to 2000 × 10^9^/L, elevated white blood cell (WBC) count up to 29 × 10^9^/L, and low hemoglobin (Hgb) concentration, 7.7 g/L. Colonoscopy and gastroscopy were performed to exclude bleeding from gastrointestinal tract. Abdominal ultrasound scan and chest X-ray did not reveal any significant pathologies. Markers of neoplasm (CEA, CA19-9, PSA) were also negative. Erythrocyte sedimentation rate (ESR) and C-reactive protein (CRP) were within normal range. Transfusion of 5 units of red blood cells concentrate was done. Morphology test a month later revealed the following: PLT 2900 × 10^9^/L, Hgb 5.1 g/L, and WBC 27 × 10^9^/L. The patient was transfusion dependent till his referral to our department.

On admission he presented with generally good condition, having only mild tinnitus and chest pain CCS (Canadian Cardiovascular Society score) II/III. His medical history contained hypertension. On physical examination the patient was pale, but fit. There was no lymphadenopathy; liver and spleen were not enlarged. Results of biochemical investigations on admission were as follows: PLT 2195 × 10^9^/L, hemoglobin concentration 6.1 g/dL, mean cell volume (MCV) 85.5 fl, WBC 24 × 10^9^/L, and differential count 56% of lymphocytes, 40% of segmented neutrophils, and 4% of monocytes and an absolute lymphocyte count of 13.44 × 10^9^/L. Review of the peripheral blood smear showed an increased number of typical small lymphocytes with spherical nuclei, coarse chromatin, and scanty cytoplasm; also smear cells were seen, as well as numerous platelets. Bone marrow aspiration was hypercellular, with 54% of mature lymphocytes. Evident hyperplasia of megakaryocytes was noted. Flow cytometry confirmed the diagnosis of CLL (59% of CD19+5+ cells displaying the following phenotype: CD20+CD43+CD23+CD22+CD79B+sIG-), ZAP70 being positive, with 78.5% of CD38+ cells. Trephine biopsy exhibited prominent proliferation of megakaryopoietic system and reticulin fibrosis of I and II degrees in all intratrabecular spaces. It also revealed a 30% interstitial and nodular infiltration pattern of B cells expressing phenotype consistent with the diagnosis of CLL. Inlets of granulopoiesis and erythropoiesis were normal. No excessive blast count was seen. Molecular assay did not detect JAK2V617F mutation. RT-PCR technique did not reveal bcr/abl fusion gene. Cytogenetics detected normal male karyotype (46,XY) in 27 metaphases. FISH analysis of peripheral blood revealed interstitial deletion of 13q14.3. No deletion of TP53 (17p13.1) and ATM gene (11q22.3) and no trisomy of 12 chromosome were found. Biochemistry showed that elevated potassium concentration of 6 mmol/L (repeatedly), reticulocyte count of 5.29%, and slightly increased LDH activity (199 IU/L) and direct and indirect Coombs tests were negative, serum haptoglobin concentration was below normal range (0.0765 g/L), and 2micro-globulin was normal (2044 *μ*g/L). Quantitative immunoglobulin test revealed the following: significantly decreased IgG (4.4 g/L), with normal IgM and IgA concentrations. Fibrinogen level was normal (2.91 g/L). Serum iron level and iron binding capacity were normal. Coagulation parameters were within normal range. Troponin I test was done to exclude any cardiac event. ECG tracing was within normal range. Echocardiography revealed normal left ventricle ejection fraction (50%), with normal diameter of all heart cavities and no significant valvular flow disorders.

In this case the diagnosis of secondary thrombocytosis was highly unlikely due to extremely high platelet count. Nonetheless, reactive thrombocytosis was excluded based on no symptoms and signs of infection, normal CRP, ESR, and negative markers of neoplasms. Other myeloproliferative disorders and iron deficiency were also ruled out. To sum up, after exclusion of reactive thrombocytosis the ET diagnosis was confirmed based on the World Health Organization (WHO) criteria (2008) [9]. Simultaneously, the patient was diagnosed with coexisting B cell CLL Rai 0 Binet A according to the WHO and International Workshop on Chronic Lymphocytic Leukemia (IWCLL) criteria [10].

The “watch and wait” strategy was applied to CLL. Due to low hemoglobin concentration RBCs concentrate was transfused. The patient was started on prednisone (1 mg/kg) with tapering dose. He responded with hemoglobin level of 8.9 g/dL and became transfusion independent. We also supplemented the immunoglobulins (Sandoglobin 12 g i.v.). To lower the elevated platelet count we decided to start the treatment with hydroxyurea at dose 2.5 g/day that resulted in platelet count decrease to 741 G/L. The patient was observed in our outpatient clinic and 3 months later his general condition was fine and his platelet count was 220 × 10^9^/L (hydroxyurea dose was reduced to 1 g/day). His CLL is in the indolent phase and is being monitored without therapeutic intervention.

## 3. Discussion

Chronic lymphocytic leukemia is recognized as the most common leukemia type in Europe and in Northern America. It is also known that the incidence rate of secondary malignancies is much higher than in the normal population [1]. It is worth stressing that the coexistence of MPNs and CLL is much less common than with other neoplasms, especially solid cancers [1, 2]. Possible pathogenetic mechanisms might include independent proliferation of two distinct cell lines, bilineage development of a common pluripotent stem cell proliferation, or an accidental situation [11].

Considering Philadelphia positive MPNs, namely, chronic myelogenous leukemia (CML), interesting data was published by Bhagavathi et al. [12] who presented a case series of concomitant CML and CLL patients. In the majority of them CLL preceded the diagnosis of CML (10 cases), simultaneous occurrence of the two malignancies was described in 6 cases, and only in 2 cases CLL had been diagnosed after CML diagnosis was set [12]. In general, male predominance was reported. The time interval to the onset of the secondary neoplasm ranged between 2 and 84 months. Most CML patients were treated with chemotherapeutic agents (such as hydroxyurea, busulphan, and interferon) because almost all of the cases are derived from preimatinib era. Only one case presented by the authors of this report was treated with imatinib [12]. Some CLL patients were also reported to be treated with chlorambucil, fludarabine, vincristine, or radiotherapy. Chemotherapeutic drugs can be the factors that might have contributed to the development of secondary neoplasm. Analyzing triggering pathogenetic mechanisms in this group of patients might be misleading then.

Among Philadelphia negative MPNs coexistent with CLL most cases include polycythemia vera and essential thrombocythemia, with only few cases of primary myelofibrosis reported. The largest review was presented by Chintapatla et al. [13] who described a case of a 57-year-old male who developed CLL after being treated for JAK2V617F negative ET with anagrelide. They also reviewed the case reports published previously. In most patients ET either preceded CLL (4 cases) or was diagnosed simultaneously (4 cases); in only one case CLL was the initial diagnosis [13]. The age at diagnosis ranged from 57 to 82 years, with male predominance. The time interval to developing secondary malignancy was 3 to 8 years. In majority of cases JAK2V617F and ZAP-70 status were unknown, since no reasonable conclusions can be drawn whether they might serve as risk stratification markers. Interestingly, other authors [14] also reported a case of CLL developing after anagrelide treatment of JAK2V617F positive ET. Vannucchi et al. [4] provided interesting retrospective analysis of more than 877 patients diagnosed with MPN in the period from 1980 to 2008. 4.5% of all patients was found to develop secondary lymphoproliferative disorder. Two cases of JAK2V617F positive ET and another 2 cases with PV were reported during 3-year follow-up. The authors concluded that the risk of secondary lymphoproliferative disorder is significantly increased in MPN patients compared to the general population (the cumulative risk at 5 and 10 years being 1 and 3%, resp.), particularly in males. They found that the risk was higher in JAK2V617F positive patients. In conclusion, they indicated that MPN patients presented a 12-fold risk of developing CLL [4].

Tabaczewski et al. [6] reported cases of CLL coexistent with JAK2 V617F positive essential thrombocythemia and set the hypothesis that during the pre-JAK2 phase of the stem cell development, an initial genetic hit occurred that predisposed the pluripotent stem cell to further genetic mutations and then stem cells differentiated into myeloid and lymphoid lineages. Further on the authors hypothesized that genetic instability may lead to JAK2 acquisition within the myeloid lineage. Moreover, the clonal evolution of B cell lineage early in the ontogenesis process may result in B cell clonal disorder, for example, CLL [6]. That hypothesis may explain why JAK2 mutation was not detected in B cell lineage [6]. Other authors [15] found no 13q14 deletion in megakaryocytes (on the contrary to B cells) that confirmed that lymphocytes and megakaryocytes did not come from the same line in the reported case of simultaneous occurrence of CLL and ET. Also Gargallo et al. [5] evaluated CLL patients coexistent with CLL and found out that the two malignancies arose from distinct clones. But still the pathogenesis remains unclear. The question as to whether these are two distinct clonal hematologic disorders is often raised.

The first systematic retrospective study was performed in 2011 by GIMEMA group [3]. In this study 46 cases of CLL and myeloproliferative neoplasms coexistence diagnosed during a 25-year-period were presented: 18 with essential thrombocythemia, 10 with polycythemia vera, 9 with chronic myelogenous leukemia, 6 with primary myelofibrosis (PMF), and 3 cases of unclassifiable myeloproliferative/myelodysplastic neoplasm, unclassifiable (MPN/MDS, UC). In the presented series of patients, more than 82% were stage A according to Rai classification of CLL, no B or C stage was detected and the rest of the patients fulfilled the criteria of MBL (monoclonal B cell monocytosis). Based on that report the incidence of MPN and CLL coexistence is calculated to be 1%. The results indicated that patients with concomitant CLL and MPN usually present indolent CLL course with good prognostic factors (mutated immunoglobulin variable (IgVH) region in 70% of cases, negativity of CD38 and Zap-70 expression in 78.5% and 71%, respectively, and a low-risk FISH karyotype (normal, +12, or del13q14) in 92.8% of the evaluable cases). Interestingly, none of MBL patients evolved into CLL and only 15% of CLL patients experienced progression of the disease that required chemotherapeutic treatment [3]. In the vast majority of cases MPN was detected in patients who had not been treated with chemotherapy for CLL. The authors conclude that a stringent hematologic follow-up in MPN patients allowed an early diagnosis of lymphoproliferative disorder. That is consistent with other data [16, 17].

Primary myelofibrosis coexisting with CLL is a very rare association. CLL may precede or follow PMF, but extremely rarely may also be diagnosed simultaneously [3, 9, 18]. Secondary myelofibrosis occurs in 20–30% of CLL cases, usually with no clinical manifestation. Marrow fibrosis may be caused by the secretion of particular cytokines that stimulate the growth and proliferation of fibroblasts [8]. Authors report male predominance with median age of 66 years in the cases of concomitant PM and CLL occurrence [18]. In majority of cases PM is the initial diagnosis. Still, the pathomechanism is unclear.

## 4. Conclusions

There is still confusion whether myelo- and lymphoproliferative disorders originate from the same cell or there are distinct entities coexisting or following one another. Most of the studies presented above indicate that there is a distinct genetic entity of both disorders [6, 8]. Contrary results can be derived from other publications [3, 14].

Chronic lymphocytic leukemia was reported to be the triggering factor of secondary malignancy development mainly due to impaired immune surveillance in those patients [2]. Theoretically, the leukemogenic effect of the chemotherapy used for CLL may be also of importance. But most authors report that MPNs were detected in patients who were in stage A/0 and had not been treated for CLL before [4, 8, 18].

Due to very sporadic simultaneous occurrence of myelo- and lymphoproliferative neoplasms, further multicenter studies are needed to assess the epidemiology and pathogenesis of these disorders. Further work involving comparison of the genetic profiles may provide valuable information concerning molecular pathogenesis.
